# Protective Role of Toll-like Receptor 3-Induced Type I Interferon in Murine Coronavirus Infection of Macrophages

**DOI:** 10.3390/v4050901

**Published:** 2012-05-24

**Authors:** Liudmila Mazaleuskaya, Rogier Veltrop, Nneka Ikpeze, Julio Martin-Garcia, Sonia Navas-Martin

**Affiliations:** 1 Department of Microbiology and Immunology, Drexel University College of Medicine, 245 North 15th Street, Philadelphia, PA 19102, USA; Email: lm429@drexel.edu (L.M.); rogierveltrop@hotmail.com (R.V.); nci25@drexel.edu (N.I.); julio.martin-garcia@drexelmed.edu (J.M.-G.); 2 Pharmacology and Physiology Graduate Program, Drexel University College of Medicine, 245 North 15th Street, Philadelphia, PA 19102, USA; 3 Center for Molecular Virology and Translational Neuroscience, Institute for Molecular Medicine and Infectious Disease, Drexel University College of Medicine, 245 North 15th Street, Philadelphia, PA 19102, USA

**Keywords:** murine coronavirus, macrophage, TLR, poly I:C, type I IFN, antiviral state

## Abstract

Toll-like Receptors (TLRs) sense viral infections and induce production of type I interferons (IFNs), other cytokines, and chemokines. Viral recognition by TLRs and other pattern recognition receptors (PRRs) has been proven to be cell-type specific. Triggering of TLRs with selected ligands can be beneficial against some viral infections. Macrophages are antigen-presenting cells that express TLRs and have a key role in the innate and adaptive immunity against viruses. Coronaviruses (CoVs) are single-stranded, positive-sense RNA viruses that cause acute and chronic infections and can productively infect macrophages. Investigation of the interplay between CoVs and PRRs is in its infancy. We assessed the effect of triggering TLR2, TLR3, TLR4, and TLR7 with selected ligands on the susceptibility of the J774A.1 macrophage cell line to infection with murine coronavirus (mouse hepatitis virus, [MHV]). Stimulation of TLR2, TLR4, or TLR7 did not affect MHV production. In contrast, pre-stimulation of TLR3 with polyinosinic-polycytidylic acid (poly I:C) hindered MHV infection through induction of IFN-β in macrophages. We demonstrate that activation of TLR3 with the synthetic ligand poly I:C mediates antiviral immunity that diminishes (MHV-A59) or suppresses (MHV-JHM, MHV-3) virus production in macrophages.

## 1. Introduction

Coronaviruses (CoVs), a genus in the Coronaviridae family, order Nidovirales, are emerging RNA pathogens of many animal species, including humans [1]. Currently there are no approved treatments or completely successful vaccines against CoV infections. Mice infected with different strains of Mouse Hepatitis Virus (MHV), the prototype of betacoronaviruses, provide animal models for human diseases. The neurotropic strains, MHV-JHM and MHV-A59, are commonly used to study viral encephalitis and virus-induced chronic demyelination, respectively [2]. MHV-A59 also triggers mild to moderate hepatitis. The highly hepatovirulent strain MHV-3 provides a model of fulminant viral hepatitis [3]. The first few days after infection with MHV are characterized by a strong innate immune response with infiltration of macrophages, neutrophils, and natural killer cells to the site of infection. It is widely documented that the host immune response plays a dual role in CoV infection. On the one hand, it limits virus spread and replication and initiates adaptive immunity; on the other hand, it triggers overproduction of cytokines and chemokines, thus contributing to the severity of the disease [2,3,4,5,6]. Macrophages are productively infected by murine CoVs [7,8,9] and represent the largest group of innate immune cells that infiltrate the central nervous system (CNS) after infection with neurotropic MHV strains [4] and the lungs of patients infected with severe acute respiratory syndrome (SARS)-CoV [10]. 

The adaptive immune response that occurs during CoV infection is well characterized [3,5], but our understanding of the interaction of CoVs with the innate immune system of the host is still emerging [4,11]. Type I interferon (IFN) (IFN-α and IFN-β) is crucial for the control of MHV infection *in vivo* [12,13,14]. In most cell lines, murine CoVs are poor inducers of type I IFN and are barely sensitive to pretreatment with IFN [15]. In primary cells, however, MHVs trigger IFN-α in plasmacytoid dendritic cells (pDCs) [12] and IFN-β in macrophages [7,9] and are sensitive to pre-treatment with IFN-β in macrophages [15]. Therefore, interaction between murine CoVs and the type I IFN response depends on the cell type. The importance of type I IFN in CoV infection is highlighted by a number of countermeasures and evasion mechanisms that CoVs in general and MHVs in particular developed to suppress signaling or prevent induction of the IFN response [16,17,18].

Induction of type I IFN can occur in all nucleated cells on TLRs activation [19]. TLRs comprise a family of Pattern Recognition Receptors (PRR) that sense conserved molecular motifs of pathogens and trigger innate immunity and prime the adaptive immune response [20]. Triggering of TLRs induces complex signaling cascades initiated by the toll/interleukin-1 receptor (TIR) domain in the cytoplasmic tail of the TLR. TIR domain-containing adaptor molecules, MyD88, which is utilized by all TLRs except for TLR3, as well as TIRAP, TRIF, and TRAM (for TLR4), are recruited to the receptor and activate a complex containing IRAKs and TRAFs which signal through NF-kB leading to the expression of a variety of genes encoding pro-inflammatory cytokines, chemokines and/or type I interferons (IFNs) that orchestrate anti-bacterial and anti-viral responses [21]. In the context of RNA virus infection, TLR2, TLR3, TLR4, TLR7, and TLR8 can potentially be activated. Cell surface TLR2 and TLR4 may recognize viral structural components, whereas endosomal TLR3 and TLR7/8 may sense viral double-stranded and single-stranded RNA, respectively [19]. All of the above-mentioned TLRs were shown to induce type I IFN through activation of transcription factors and Interferon Regulatory Factors (IRFs); the magnitude of response, however, depends on the stimulus and the cell system. TLR3, TLR4 and TLR7 are known to be potent inducers of the IFN response depending on the cell type [22]. In contrast, TLR2 has been considered until recently a poor inducer of IFN response, despite triggering of TLR2 with bacteria-derived ligands induces strong pro-inflammatory cytokine response. In this regard, emerging evidence suggests that TLR2 and TLR4 activation induces pro-inflammatory cytokine and type I IFN responses from distinct sub-cellular sites: the plasma membrane and the endolysosomal compartments, respectively [23,24]. Interestingly, only a particular monocyte subset has been reported to induce type I IFN through TLR2 in response to viral ligands [25]. Once secreted, IFN-α/β act through the JAK-STAT signaling pathway that triggers an “antiviral state” and help to eliminate viral infection [19,26]. 

The ability of TLRs to trigger antiviral immunity makes them a promising target for antiviral therapeutics. Stimulation with TLR agonists has been shown to provide protection from some viral infections, such as hepatitis B virus (through TLR3, TLR4, TLR5, TLR7, or TLR9) [27], herpes simplex virus encephalitis (through TLR3) [28], lethal influenza virus (through TLR3 or TLR9) [29], HIV strains Bal and Jago (through TLR3) [30], and hepatitis C virus (through TLR7) [31]. This study was undertaken to assess the effect of ligand-mediated, TLR activation of macrophages on their susceptibility to infection with murine CoV. We profiled TLR2, TLR3, TLR4, and TLR7 agonists (heat-killed Listeria monocytogenes (HKLM), poly I:C, lipopolysaccharide (LPS), and imiquimod, respectively) and observed differential effects of these ligands on MHV production in macrophages. Of all the ligands tested, only the triggering of TLR3 with poly I:C induced a strong antiviral response. Mechanistically, the antiviral effect of poly I:C was promoted in a type I IFN-dependent manner. 

## 2. Results and Discussion

### 2.1. Triggering of TLR3, but not TLR2, TLR4, or TLR7 Inhibits Virus Production in MHV-Infected Macrophages

Ligand-mediated activation of TLRs has been reported to affect the infectivity of various viruses [27,30,31,32,33,34,35]. The potential immunomodulatory and antiviral effects of triggering TLRs against CoV infections in macrophages have not yet been investigated. Macrophages are antigen-presenting cells that express TLRs and play a pivotal role in CoV pathogenesis. The goal of this study was to investigate the effect of activation of TLR2, TLR3, TLR4, or TLR7 with selected ligands on macrophage susceptibility to infection with murine coronavirus. We chose these TLRs on the basis of their potential role in the recognition of MHV by macrophages. TLR2 has been shown to recognize MHV-3 in peritoneal macrophages [8]; TLR4 has been implicated in protection and pathogenesis in MHV-1-induced respiratory infection [36]. Despite the fact that TLR3 is a sensor of dsRNA and could sense CoV intermediate replicative forms in infected cells, its role in the recognition of CoVs or in their pathogenesis has not yet been established. TLR7 senses MHV-A59 in pDCs [12].

**Figure 1:**
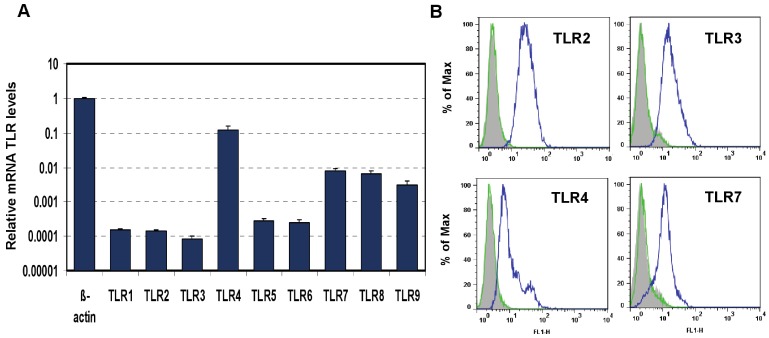
Expression of Toll-like Receptors (TLRs) in J774A.1 murine macrophages. (**A**) J774A.1 cells were profiled for TLR1-9 gene expression by quantitative real-time polymerase chain reaction (PCR) using predeveloped TaqMan Gene Expression assays (AppliedBiosystems). Expression levels of target genes were normalized to the housekeeping gene β-actin (ΔCt). Relative gene expression values were calculated based on the ΔΔCt method, with data for all samples analyzed against the mean value of four replicates; (**B**) Expression of cell surface TLR2 and TLR4, and intracellular TLR3 and TLR7 in naïve J774A.1 cells was analyzed by flow cytometry (FACS) using standard protocols. Empty, dashed and blue histograms represent only cells (no antibodies), isotype antibody controls, and TLR expression in 774A.1 cells, respectively.

First, we developed an *in vitro* model suitable for this study. The mouse macrophage cell line J774A.1 was profiled for TLR1-9 gene expression by quantitative real-time polymerase chain reaction (PCR) using predeveloped TaqMan Gene Expression assays (AppliedBiosystems Life Technologies Corp, Carlsbad, CA) (Figure 1A). Expression levels of target genes were normalized to the housekeeping gene β-actin (ΔCt). Gene expression values were calculated based on the ΔΔCt method, with data for all samples analyzed against the mean value of four replicates. TLR4 showed a 10-fold greater expression than that of TLR7 and TLR 9 (Student’s t test, *p* < 0.05), and the latter two had a more than 10-fold greater expression than TLR1, 2, 3, 5, and 6 (Student’s t test, *p* < 0.05). TLR4 and TLR3 transcripts were expressed to the highest and lowest levels, respectively (Student’s t test, *p* < 0.05). The expression of TLR2, TLR3, TLR4, and TLR7 proteins was analyzed using flow cytometry (FACS). As shown in Figure 1B, FACS data demonstrated robust expression of cell-surface TLR2 and TLR4 and intracellular TLR3 and TLR7 in naïve J774A.1 cells. 

**Figure 2:**
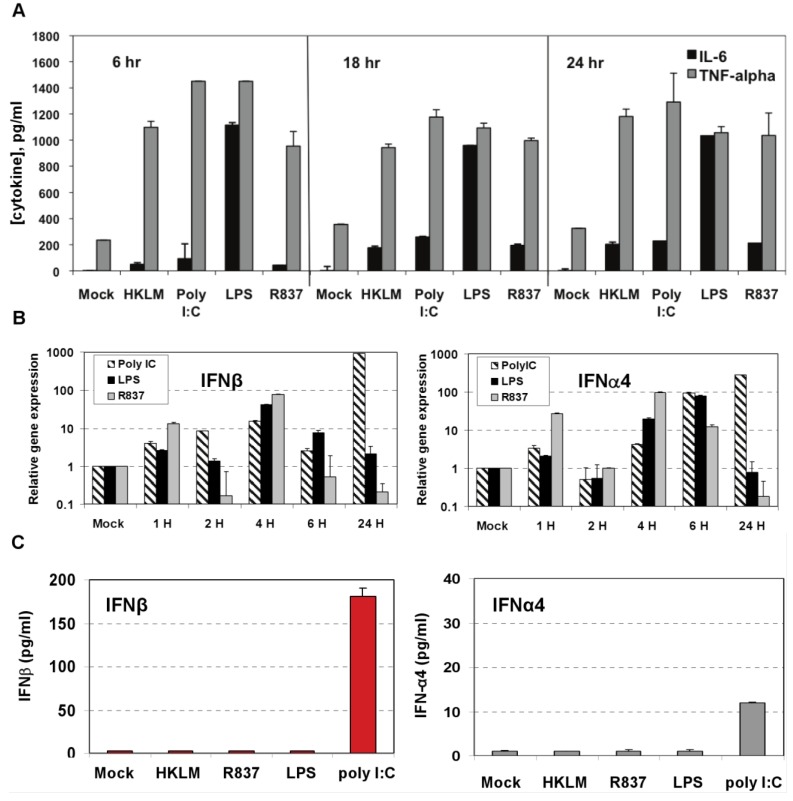
Induction of proinflammatory cytokine response and type I IFN after triggering with ligands specific to TLR2-TLR4, and TLR7 in J774A.1 cells. (**A**) J774A.1 macrophages were stimulated with 10^8^ cells/mL HKLM (TLR2), 1 μg/mL poly I:C (TLR3), 5 μg/mL LPS (TLR4), or 5 μg/mL imiquimod (R837) (TLR7) for 6, 18 and 24 h. Cell-free supernatants were assessed for the production of IL-6 and TNF-α using the enzyme-linked immunosorbent assay (ELISA). Error bars represent the standard error of the mean of two replicates; (**B**) Real-Time PCR of type I IFN gene expression in TLR stimulated J774A.1 macrophages. J774A.1 was profiled for IFNβ and IFNα4 gene expression by quantitative real-time PCR using predeveloped TaqMan Gene Expression assays (AppliedBiosystems). Expression levels of target genes were normalized to the housekeeping gene 18S rRNA (ΔCt). Gene expression values were calculated based on the ΔΔCt method, with data for all samples analyzed against the mean value of four replicates. Error bars represent the standard error of the mean of two independent experiments, each done in duplicate; (**C**) Type I IFN production in TLR2-4 and TLR7 activated J774A.1 cells. J774A.1 macrophages were stimulated with 10^8^ cells/mL HKLM (TLR2), 0.25 μg/mL poly I:C (TLR3), 5 μg/mL LPS (TLR4), or 5 μg/mL imiquimod (R837) (TLR7) for 6 h. Supernatants collected 6 h after TLR stimulation were assessed for IFN-α and IFN-β production using ELISA. Error bars represent the standard error of the mean of two independent experiments, each done in duplicate.

Next we determined whether TLR2, TLR3, TLR4, and TLR7 are functional in J774A.1 cells. Activation of the cells with a TLR2 ligand (HKLM, 10^8^ cells/mL), a TLR3 agonist (poly I:C, 1 μg/mL), a TLR4 ligand (LPS, 5 μg/mL), or a TLR7 agonist (imiquimod (R837), 5 μg/mL) for 6, 18, and 24 h resulted in the robust production of IL-6 and TNF-α (Figure 2A). This result indicates that, in J774A.1 macrophages, TLR2-4 and TLR7 are fully functional and signal with cytokine secretion after stimulation. We assessed type I IFN mRNA induction in TLR-activated J774A.1 macrophages. Expression of IFN-α and IFN-β genes was up regulated by poly I:C, LPS, and R837 ligands in J774A.1 cells with different kinetics (Figure 2B). LPS- induced IFN-β and IFN-α4 mRNAs peaked at 4 h and 6 h post-stimulation, respectively. R837- induced IFN-β and IFN-α4 mRNAs peaked at 4 h post-stimulation. The induction of type I IFN gene expression after LPS and R837 was not sustained (in contrast to IFN-β gene expression after Poly I:C stimulation). 

IFN-α4 and IFN-β levels were determined by ELISA in cell-free supernatants collected after 6 h of prestimulation with HKLM (10^8^ cells/mL), LPS (5 μg/mL), R837 (5 μg/mL), and poly I:C (0.25 μg/mL). Interestingly, activation of TLR3 but not of TLR2, TLR4 or TLR7 triggered robust production of IFN-β following pre-stimulation for 6 h in macrophages (Figure 2C). Similar to IFN-β, IFN-α4 was secreted only in TLR3-activated cells, albeit to a much lesser degree (Figure 2C). These contrasting results suggest that type I IFN response may be regulated differentially on TLR3 stimulation at the post transcriptional level in J774A.1 cells. Their lack of IFN secretion (as measured by ELISA) in response to stimulation with the bacterial ligands HKLM and LPS is somewhat unpredicted and deserves further investigations. In addition, although it is well established that type I IFN response against RNA viruses is mainly mediated by pDCs via a TLR7-dependent pathway, the role of TLR7 in macrophage activation remains poorly understood. In this regard, the absence of IFN-β secretion after R837 stimulation of TLR7 in J774A.1 cells may suggest differences in the regulation of the TLR7 pathway and/or its effectors between macrophages and pDCs. We are currently investigating the role of macrophage TLR7 in the antiviral response against RNA viruses.

Furthermore, MHVs have been shown to productively infect J774A.1 cells [37]. By combining these results, we established a valid *in vitro* model in which to investigate the effect of triggering TLRs with selected ligands on MHV infectivity in macrophages.

**Figure 3:**
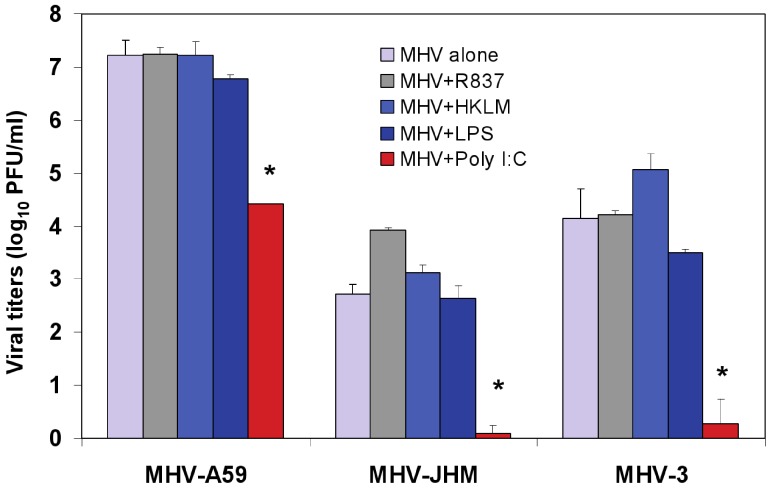
Effect of triggering TLR2, TLR3, TLR4, and TLR7 on virus production in MHV-infected J774A.1 macrophages. J774A.1 cells were prestimulated with TLR ligands for 6 h, infected with MHV-A59, MHV-JHM or MHV-3 (1 MOI) by adsorption for 1h in the absence of the ligands, and activated for up to 18 h p.i. with the appropriate TLR agonist. TLR ligands were used as follows: HKLM (TLR2) at 10^8^ cells/mL; LPS (TLR4) at 5 μg/mL; Imiquimod (R837) (TLR7) at 5 μg/mL. Poly I:C (TLR3) was tested at a range of concentrations (0.25, 0.5, and 1.0 μg/mL). Because poly I:C triggered a comparable effect on MHV production at all concentrations (data not shown), viral titers at 0.25 μg/mL were included in the plot. Cells incubated with the basal medium before and during infection served as a negative control for the effect of TLR triggering on virus production. MHV titers were assessed in cell-free supernatants using a plaque assay on L2 fibroblasts. The data shown are the mean viral titers of three independent experiments, each done in duplicate ± standard deviation (*p value relative to virus alone, *p* < 0.001 Student’s *t* test).

To test whether treatment with the TLR ligands HKLM, poly I:C, LPS, and R837 affected the replication of MHV, J774A.1 cells were prestimulated with the appropriate ligand at the above-mentioned concentrations for 6 h; and cells were infected with MHV-A59, MHV-JHM, or MHV-3 at a multiplicity of infection (MOI) of 1 by adsorption for 1 h in the absence of the ligands and stimulated again for up to 18 h postinfection (p.i.) with the appropriate TLR agonist. Therefore, there were two challenges with TLR ligands: one before and one after virus adsorption. Activation of macrophages with TLR2, TLR4 and TLR7 did not noticeably affect MHV production in J774A.1 macrophages (Figure 3). Conversely, the triggering of TLR3 with poly I:C significantly inhibited MHV-A59, MHV-JHM, and MHV-3 production relative to virus alone (Student’s t test, *p* = 0.0001; Figure 3). Complete suppression was observed only in poly I:C-treated MHV-JHM- and MHV-3-, infected macrophages, although all MHV strains showed a dramatic 3-log reduction in virus production. The lack of complete suppression in MHV-A59 infected cells could be explained by the ability of MHV-A59 to grow to higher titers (3–4 log) than MHV-JHM and MHV-3 in macrophages. Additionally, these results may also suggest that MHV-A59 counteracts the TLR3 pathway in J774A.1 macrophages. Indeed, our data shows that TLR3-mediated, IFN-β secretion is significantly reduced in MHV-A59-infected macrophages (Figure 6). Interestingly, poly I:C triggered comparable antiviral effect regardless of its concentration (0.25, 0.5, and 1.0 μg/mL; data not shown). It will be of interest to determine the minimal antiviral concentration of poly I:C in future experiments. The optimal concentration range for poly I:C was selected based on the highest rate of cytokine production (IL-6 ELISA) and minimal cytotoxicity (LDH cytotoxicity assay) in J774A.1 macrophages activated with poly I:C at various doses (data not shown).

Collectively, these data demonstrate that, depending on the receptor, ligand-mediated TLR stimulation exerts differential effects on MHV production. Triggering TLR3 with poly I:C, but not activation of TLR2, TLR4, or TLR7 with their respective ligands, impairs MHV replication in macrophages with a comparable magnitude of suppression of viral titers for MHV-A59, MHV-HJM, and MHV-3 strains. Given that all four TLR ligands induced strong IL-6 and TNF-α proinflammatory responses (Figure 2A), we concluded that the inability of TLR2, TLR4 and TLR7 agonists to protect macrophages from MHV infection is not due to the lack of signaling through these receptors, rather it stems from the absence of IFN-α and IFN-β production after 6 h of stimulation with their ligands (Figure 2C). In contrast, the antiviral effect mediated by activation of TLR3 with Poly I:C is associated with a sustained transcriptional upregulation and secretion of IFN-α4 and IFN-β.

### 2.2. TLR3 Activation with Poly I:C Inhibits MHV Production in Pre-, Post-, and Simultaneously Treated MHV-Infected Macrophages

**Figure 4:**
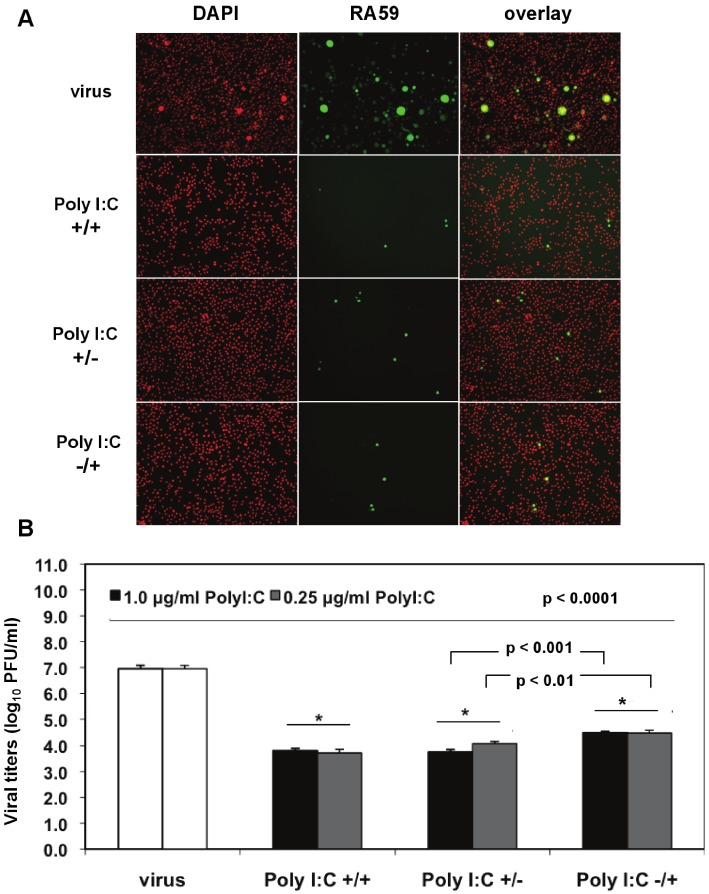
Prestimulation with poly I:C before virus adsorption is sufficient to trigger a profound antiviral effect in MHV-infected macrophages. (**A**) J774A.1 macrophages were prestimulated with poly I:C for 6 h and coactivated during RA59-GFP (1 MOI) infection for 18 h postadsorption at concentrations of 0.25 and 1.0 μg/mL of the TLR3 ligand. Cells were treated as follows: (1) poly I:C prestimulated only (poly I:C +/−); (2) poly I:C coactivated only after virus adsorption (poly I:C −/+); and (3) poly I:C-treated before and after virus adsorption (poly I:C +/+). Unstimulated but infected macrophages served as a negative control for poly I:C antiviral effect. RA59-GFP infection was visualized at the original magnification x100. The data shown are representative images of two independent wells for cells treated with 0.25 μg/mL poly I:C. Original magnification x100. (**B**) RA59-GFP titers were assessed in cell-free supernatants from (**A**) using a plaque assay on L2 fibroblasts. Error bars represent the standard error of the mean of two replicates (* *p* value relative to virus alone; other *p* values relative to Poly I:C-pre-stimulated cells only, Student’s *t* test).

We investigated the optimal conditions for poly I:C antiviral effects in J774A.1 macrophages infected with a recombinant MHV-A59 expressing the GFP protein (RA59-GFP) (1 MOI) and treated as follows: (1) prestimulated with poly I:C, with no drug present during infection (poly I:C +/−); (2) treated with poly I:C only after virus adsorption (poly I:C −/+); (3) treated with poly I:C before and after virus adsorption (poly I:C +/+). The TLR3 ligand was used at concentrations of 0.25 to 1.0 μg/mL for 6 h of prestimulation and/or 18 h p.i. A profound suppression of GFP expression in cells stimulated with 0.5 μg/mL poly I:C was observed with all of the above-mentioned treatments relative to infected macrophages in the absence of the drug (Figure 4A). Thus, a single challenge with the TLR3 ligand before or after virus adsorption was sufficient to trigger a robust antiviral effect comparable to cells challenged with poly I:C twice. To determine the level of MHV production, released virus was quantified by plaque assay in cell-free supernatants from macrophages stimulated with 0.25 and 1.0 μg/mL poly I:C as above and in the absence of the drug (Figure 4B). Regardless of the concentration of poly I:C, the triggering of TLR3 with poly I:C resulted in a 3-log reduction in RA59-GFP titers in prestimulated and coactivated macrophages (poly I:C +/+) relative to infected cells in the absence of the drug (Figure 4B, *p* < 0.0001). Cells challenged with Poly I:C once before (Poly I:C +/−) or 1 h after MHV adsorption (Poly I:C −/+) also exhibited a significant suppression (*p* < 0.0001) of virus production comparable to that of prestimulated and coactivated macrophages (poly I:C +/+). Interestingly, the triggering of TLR3 before adsorption with MHV (poly I:C +/−) resulted in significantly lower virus production relative to coactivated macrophages (poly I:C −/+) (*p* = 0.01 and *p* = 0.001 for 0.25 and 1.0 μg/mL poly I:C, respectively), suggesting that a single challenge with poly I:C prior to infection dramatically reduces macrophage susceptibility to MHV infection. 

Taken together, these results indicate that 0.25 μg/mL of poly I:C is sufficient to trigger a profound TLR3-mediated antiviral effect and that prestimulation alone is enough to protect macrophages from infection with MHV. 

### 2.3. Poly I:C Triggers Secretion of Soluble Factors that Promote an Anti-viral Effect in MHV-Infected Macrophages

**Figure 5:**
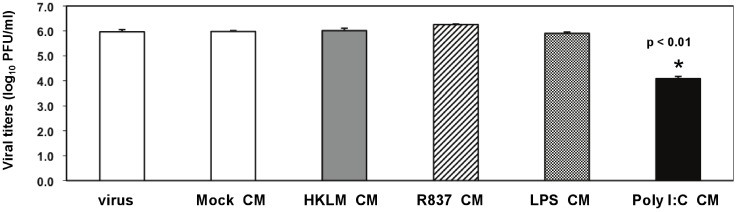
Involvement of soluble factors in the antiviral effect mediated by TLR3 in poly I:C-stimulated, MHV-infected macrophages. J774A.1 macrophages were pretreated for 3 h with the conditioned medium (CM) from macrophages prestimulated with TLR ligands for 6 h from Figure 2. CM was diluted 1:1 with the basal medium to replenish nutrients. Then cells were infected with RA59-GFP (1 MOI) for 1 h adsorption and incubated in the basal medium for up to 18 h p.i. Cells pretreated with the CM from mock cells or with fresh basal medium served as negative controls for the TLR-triggered effect. RA59-GFP titers were assessed in cell-free supernatants using a plaque assay on L2 fibroblasts. Error bars represent the standard error of the mean of two replicates (*p* value relative to cells pretreated with CM from mock, *p* < 0.01, Student’s *t* test).

To investigate the mechanism of the poly I:C-triggered antiviral effect in MHV-infected macrophages, we wanted to determine if the TLR3 ligand induced soluble factors that mediated protective immunity against CoV infection. We pretreated J774A.1 cells for 3 h with conditioned medium (CM) from macrophages prestimulated with TLR2-4 and TLR7 ligands for 6 h (Figure 2A). Next, cells were infected with RA59-GFP (1 MOI) for 1 h adsorption in the absence of TLR ligands and incubated for additional 17 h in basal medium. RA59-GFP virus titers determined by plaque assay are shown in Figure 5. As expected, CM from mock macrophages (unstimulated, uninfected) did not affect virus production. Treatment with CM from macrophages stimulated with HKLM, R837, and LPS did not affect virus production (Figure 5). Remarkably, there was a 2-log reduction in RA59-GFP titers in cells pretreated with poly I:C CM (Figure 5, *p* < 0.01) that correlated with the inhibition of GFP expression in these cells (data not shown).

Overall, these data suggest that prestimulation with poly I:C but not HKLM, LPS, or R837 triggers the production of soluble factors that further protect macrophages from infection with MHV on subsequent exposure. In addition to soluble factors, residual poly I:C in the CM may have also contributed to the antiviral effect in cells pretreated with TLR3-stimulated supernatants.

### 2.4. Activation of TLR3 but not TLR2, TLR4 or TLR7 Induces a Profound Type I IFN Response in Activated and MHV-infected Macrophages

Our data in Figure 2C demonstrated that after 6 h of stimulation with HKLM, LPS, R837, and poly I:C, IFN-β and IFN-α4 were secreted only in TLR3-activated J774A.1 cells. These results together with the antiviral effect of poly I:C (Figure 3) suggested that the protective role of TLR3 against coronavirus infection in J774A.1 macrophages may be mediated by type I IFN. To investigate the role of type I IFN in the poly I:C-mediated inhibition of murine CoV production in macrophages, we focused on MHV-A59 and MHV-JHM strains. We hypothesized that the differential effect of TLR ligands on MHV production is due to their variable ability to induce type I IFN crucial for triggering an “antiviral state” and protecting cells from virus infection [26,38]. To test this hypothesis, we assessed type I IFN production in TLR-stimulated and/or MHV-infected J774A.1 macrophages. 

**Figure 6:**
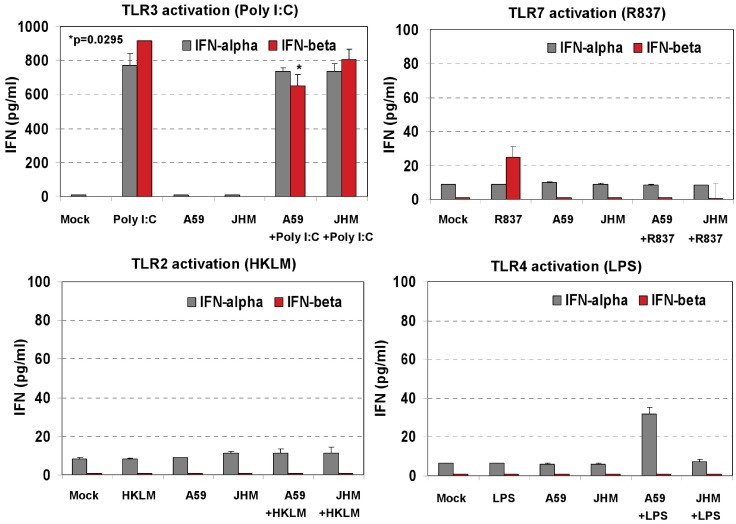
Type I IFN production in cell-free supernatants from J774A.1 macrophages stimulated with TLR ligands, MHV-infected, and/or coactivated during MHV infection. J774A.1 cells were prestimulated for 6 h with HKLM (10^8^ cells/mL), LPS (5 μg/mL), R837 (5 μg/mL), and poly I:C (0.25 μg/mL); media was removed and: (1) a second challenge of the corresponding TLR ligand (same concentrations) was added to the cells for 18 h; (2) cells were prestimulated for 6 h with the TLR ligands as above; media was removed and cells infected with MHV-A59 or MHV-JHM at 1.0 MOI for 1 h of adsorption in the absence of the ligands; after virus adsorption cells were stimulated with a second challenge of the TLR ligands using the same concentrations as during prestimulation and samples were taken at 18 h; (3) cells were not TLR activated and only infected with MHV-A59 or MHV-JHM at 1.0 MOI for 1 h of adsorption in the absence of the ligands; fresh media was added to the cells for 18 h. Non-stimulated, non-infected J774A.1 cells were used as mock control. Cell-free supernatants were taken at 18 h from TLR-activated alone, infected alone, and TLR-activated and infected and assessed for IFN-α and IFN-β production using ELISA. Error bars represent the standard error of the mean of two independent experiments, each done in duplicate; * *p* < 0.05, Student’s *t* test.

J774A.1 cells were: (1) prestimulated for 6 h with HKLM (10^8^ cells/mL), LPS (5 μg/mL), R837 (5 μg/mL), and poly I:C (0.25 μg/mL); media was removed and fresh media with the corresponding TLR ligand was added to the cells for 18 h; (2) cells were left unstimulated and only infected with MHV-A59 or MHV-JHM at 1.0 MOI for 1 h of adsorption in the absence of the ligands; fresh media was added to the cells for 18 h; (3) cells were prestimulated for 6 h with the TLR ligands as above; media was removed and cells infected with MHV-A59 or MHV-JHM at 1.0 MOI for 1 h of adsorption in the absence of the ligands; after virus adsorption cells were stimulated with a second challenge of the TLR ligands using the same concentrations as during prestimulation and samples were taken at 18 h. Non-stimulated, non-infected J774A.1 cells were used as mock control. INF-α4 and IFN-β levels were determined by ELISA in cell-free supernatants collected after 6 h of prestimulation with HKLM (10^8^ cells/mL), LPS (5 μg/mL), R837 (5 μg/mL), and poly I:C (0.25 μg/mL) (Figure 2C); and at 18 h p.i. (Figure 6).

A second challenge with poly I:C for 18 h resulted in a robust secretion of IFN-α4 and IFN-β (Figure 6). In contrast, a single challenge with poly I:C for 6 h induced lower levels of IFN-β and levels of IFN-α4 that were close to the limit of detection of the ELISA assay (Figure 2C). Such a pattern of induction of type I IFN in cells treated with dsRNA (like poly I:C) is consistent with the activation of two types of type I IFN genes, immediate-early and delayed-type genes (reviewed in ref. [26]). Immediate-early genes, mostly IFN-β and some IFN-α4 (only in murine cells), are induced by a protein-synthesis-independent pathway. Secreted IFN signals in both an autocrine and paracrine fashion through the type I IFN receptor and triggers delayed-type IFNs (including other IFN-α subtypes). Expression of the delayed-type IFN depends on *de novo* protein synthesis and results in amplification of the IFN response. Similarly, poly I:C-activated J774A.1 macrophages exhibit high levels of IFN-β and a modest secretion of IFN-α4 on induction of the immediate-early gene. Later, these cells secrete comparably high levels of both IFN-α and -β as a result of delayed-type IFN gene expression. 

MHV-A59 and MHV-JHM infection did not induce type I IFN secretion in J774A.1 macrophages as determined by ELISA. Interestingly, infection with MHV-A59 but not with MHV-JHM reduced the level of IFN-β secreted in poly I:C-treated macrophages (*p* = 0.05, Figure 6). The effect of infection with MHV on poly I:C-triggered IFN-β induction was previously assessed in 17CI-1 murine fibroblasts [39]. In that study, neither MHV-A59 nor MHV-JHM inhibited IFN-β induction after poly I:C was transfected into fibroblasts (a way to activate RIG-I and MDA5 cytoplasmic helicases but not endosomal TLR3). Thus, the ability of MHV to counteract poly I:C-induced IFN-β is cell type-specific and depends on the mode of delivery of poly I:C into the cell. Targeting RIG-I and MDA5 helicases by poly I:C transfection with a lipid carrier as reported by [39], did not result in MHV-mediated inhibition of poly I:C-induced IFN-β secretion. In contrast, our data suggest that MHV-A59 might counteract the IFN-β response when macrophages are stimulated with soluble poly I:C to trigger the TLR3 pathway. Further experiments are needed to define how MHV-A59 might counteract the TLR3 pathway in macrophages. Interestingly, MHV-A59 has been reported to develop various measures to counteract the type I IFN response [40,41,42].

Besides poly I:C, the TLR7 ligand R837 induced a modest level of secretion of IFN-β with 18 h stimulation; TLR2 and TLR4 agonists did not promote type I IFN secretion in J774A.1 macrophages as measured by ELISA (Figure 6). Overall, these findings are in agreement with our hypothesis that poly I:C triggers an antiviral effect via IFN-α/β, whereas the lack of a strong type I IFN production during TLR2, TLR4, or TLR7 signaling is responsible for uncontrolled MHV production in J774A.1 macrophages.

**Figure 7:**
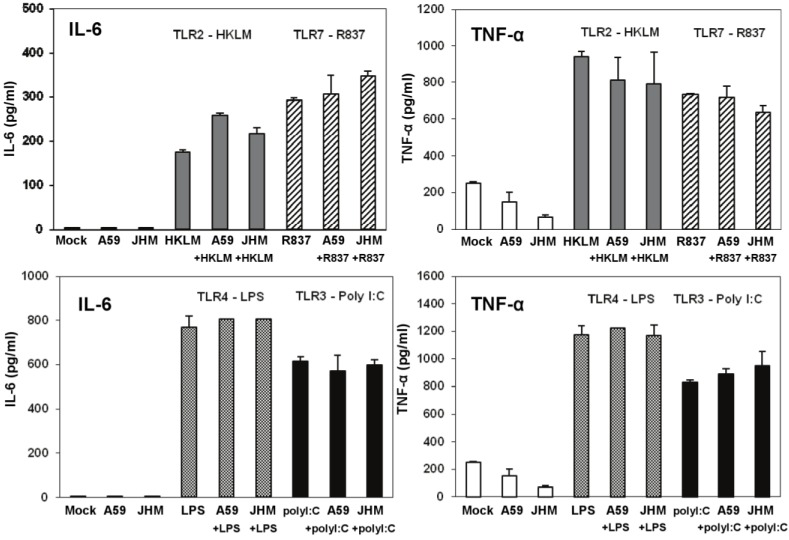
IL-6 and TNF-α production in cell-free supernatants from J774A.1 macrophages stimulated with TLR ligands, MHV-infected, and/or coactivated during MHV infection. J774A.1 cells were prestimulated for 6 h with HKLM (10^8^ cells/mL), LPS (5 μg/mL), R837 (5 μg/mL), and poly I:C (0.25 μg/mL); media was removed and: 1) a second challenge of the corresponding TLR ligand (same concentrations) was added to the cells for 18 h; 2) cells were prestimulated for 6 h with the TLR ligands as above; media was removed and cells infected with MHV-A59 or MHV-JHM at 1.0 MOI for 1 h of adsorption in the absence of the ligands; after virus adsorption cells were stimulated with a second challenge of the TLR ligands using the same concentrations as during prestimulation and samples were taken at 18 h; 3) cells were not TLR activated and only infected with MHV-A59 or MHV-JHM at 1.0 MOI for 1 h of adsorption in the absence of the ligands; fresh media was added to the cells for 18 h. Non-stimulated, non-infected J774A.1 cells were used as mock control. Cell-free supernatants were taken at 18 h from TLR-activated alone, infected alone, and TLR-activated and infected and assessed for IL-6 and TNF-α using ELISA. Error bars represent the standard error of the mean of two independent experiments, each done in duplicate.

To further rule out the potential antiviral effect of IL-6 and TNF-α in infected and co-stimulated cells, we measured proinflammatory cytokines in the same samples as above. Stimulation for 18 h resulted in a very high induction of both cytokines after LPS (a TLR ligand that based on our data does not induce antiviral effect against infection with MHV in J774A.1 macrophages) and poly I:C (albeit to a lesser extent than with LPS) (Figure 7). Overall, these data argue against the potential antiviral effects of IL-6 and TNF-α in TLR3-activated macrophages. 

TNF-α levels were reduced in MHV-A59 and MHV-JHM infected macrophages relative to mock cells (Figure 7). Although it was not the focus of the present study, the inhibitory effect of MHV on the basal TNF-α levels may be mediated through the action of the anti-inflammatory cytokine IL-10. IL-10 is a known negative regulator of TNF-α production and function in macrophages (reviewed in ref. [43]). MHV-A59 was reported to induce IL-10 in infected primary bone marrow-derived macrophages [9], therefore, one could speculate that CoVs suppress basal macrophage TNF-α secretion through triggering of IL-10. Future studies will be designed to test this hypothesis. Although TNF-α was reported to induce a strong antiviral response against various influenza strains in lung epithelial cells [44], our data demonstrated that TLR-induced TNF-α does not affect MHV production in macrophages. Collectively, these results indicate that IL-6 and TNF-α are not responsible for and do not contribute to a poly I:C-triggered antiviral effect in MHV-infected macrophages. 

### 2.5. IFN-β Mediates Poly I:C-Triggered Antiviral Response in MHV-Infected Macrophages

**Figure 8:**
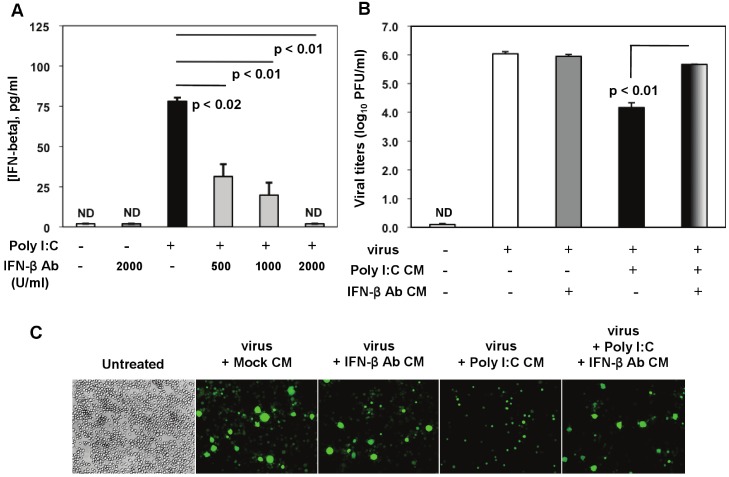
Role of IFN-β in poly I:C-triggered anti-viral response in MHV-infected macrophages. (**A**) Anti-IFN-β Ab was titrated in the presence of poly I:C in J774A.1 macrophages. Cells were activated with 0.25 μg/mL poly I:C with or without anti-IFN-β Abs at 500, 1000, 2000 U/mL. Supernatants were collected after 6 h, cleared of cell debris and assessed with IFN-β ELISA. Error bars represent the standard error of the mean of two replicates (p value relative to cells activated with poly I:C alone, Student’s *t* test). ND, not detected; (**B**) RA59-GFP titers were assessed in cell-free supernatants from (**C**) using a plaque assay on L2 fibroblasts. Error bars represent the standard error of the mean of two replicates (p value relative to cells pre-treated with Poly I:C conditioned medium, Student’s *t* test); (**C**) J774A.1 macrophages were pretreated for 3 h with the conditioned medium (CM) from the anti-IFN-β Ab titration assay in (**A**). CM was diluted 1:1 with the basal medium to replenish nutrients. Then cells were infected with RA59-GFP (1 MOI) for 1 h adsorption and incubated in the basal medium for up to 18h p.i. Cells pretreated with the CM from mock cells or fresh basal medium (untreated) served as negative controls for the poly I:C+/-anti-IFN-β Ab-triggered effect. RA59-GFP infection was visualized at the original magnification x100. The data shown are representative images of two independent wells per treatment (*p* values, Student’s *t* test).

Considering that soluble factors mediate the poly I:C-triggered antiviral effect (Figure 5) and that poly I:C potently induces type I IFN before and during virus infection (Figure 2C and Figure 7), we further confirmed the role of IFN-β in TLR3-triggered MHV suppression in macrophages (Figure 8A-C). We focused on IFN-β because unlike IFN-α, IFN-β was profoundly induced in prestimulated macrophages at 6 h poststimulation (Figure 2C), and CM from macrophages treated with poly I:C for 6 h exhibited inhibition of MHV-A59 production sufficient to reduce viral infection (Figure 5). Titration of anti-IFN-β antibody (Ab) was done to establish the optimal Ab concentration for neutralization of poly I:C-stimulated IFN-β. J774A.1 macrophages were stimulated with 0.25 μg/mL poly I:C in the presence or absence of anti-IFN-β Ab at various concentrations. We chose 0.25 μg/mL poly I:C because prestimulation with such a low concentration of the TLR3 ligand was sufficient to promote a strong antiviral effect (Figure 3). Poly I:C-triggered IFN-β was significantly reduced by 500 U/mL to 1000 U/mL of the IFN-β neutralizing Ab, and it was suppressed in the presence of a higher concentration (2000 U/mL) (Figure 8A). 

In neutralization assays, J774A.1 cells were pretreated for 3 h with CM from (1) mock (non-stimulated, non-infected macrophages); (2) CM from macrophages treated with 2000 U/mL IFN-β neutralizing Ab (IFN-β Ab CM); (3) activated with 0.25 μg/mL of poly I:C (poly I:C CM); and (4) CM from activated macrophages with 0.25 μg/mL of poly I:C in the presence of 2000 U/mL anti-IFN-β neutralizing Ab (poly I:C + IFN-β Ab CM). After stimulation, cells were infected with RA59-GFP (1 MOI) by adsorption for 1 h and incubated in fresh medium for up to 18 h p.i. CM from mock or IFN-β Ab-treated macrophages did not affect MHV-A59 virus production in these cells (Figure 8B,C). As expected on the basis of our previous results, pretreatment with poly I:C-conditioned medium resulted in a drastic reduction in RA59-GFP expression and in a 2-log reduction in MHV production in infected macrophages (Figure 8B,C). Importantly, MHV-A59 infection was significantly restored (*p* = 0.01) in J774A.1 cells incubated with the supernatant from macrophages activated with poly I:C in the presence of the IFN-β neutralizing polyclonal Ab (poly I:C + IFN-β Ab CM) (Figure 8B,C). This result indicates that IFN-β is a crucial mediator in the antiviral response against MHVs elicited by triggering TLR3 with poly I:C in macrophages.

Murine CoVs are sensitive to pretreatment of macrophages with recombinant IFN-β [15]. In the present study prestimulation of J774A.1 macrophages with poly I:C, a potent type I IFN inducer, resulted in a strong IFN-β response that triggered antiviral immunity and protected macrophages from MHV infection before and after virus adsorption. In support of our data, a poly I:C analog Ampligen^TM^ (Poly I:Poly C_12_U) was successfully tested in SARS-CoV animal models [45,46]. BALB/c mice were treated with Ampligen^TM^ intraperitoneally (i.p.) 4h before SARS-CoV infection and then the virus titers in the lungs were assessed 3 days after virus exposure. SARS-CoV titers in the lungs were below the limit of detection suggesting that poly I:poly C_12_U completely protected mice from viral infection [45]. In a different study, Ampligen^TM^ was given intraperitoneally to BALB/c mice 16 h before they were infected with the mouse-adapted SARS-CoV strain v2163. Treated mice exhibited complete survival, suppressed virus titers in the lungs, significantly reduced lung scores and weight loss [46]. These studies did not investigate the mechanism of the Ampligen^TM^-triggered antiviral effect in SARS-CoV-infected mice; type I IFN, however, was proposed as a mediator of antiviral immunity. Ampligen^TM^ is indeed a potent type I IFN inducer that acts through TLR3 [47] and triggers protection from HIV [48,49], coxsackie virus [50], Punta Toro virus [47], Venezuelan equine encephalitis virus [51], and influenza virus [52] infections. Overall, our data demonstrates that TLR3 triggered type I IFN inhibits murine CoV infection of macrophages.

## 3. Experimental Section

### 3.1. Cells

J774A.1 murine macrophages (ATTC, TIB-67) were cultured in Dulbecco’s modification of Eagle’s medium (DMEM) (cellgro, Mediatech, Manassas, VA, USA) supplemented with 10% v/v heat-inactivated fetal bovine serum (hiFBS) (Hyclone, Thermo Scientific, Rockford, IL, USA), 100 U/mL penicillin (cellgro), 100 μg/mL streptomycin (cellgro), 1.5 g/L sodium bicarbonate (BioWhittaker, Lonza, Walkersville, MD, USA) and 4 mM glutamine (cellgro). Cells were incubated in a humidified atmosphere at 37 °C with 5% CO_2_.

### 3.2. Viruses, Viral Infections, Plaque Assays

The dualtropic MHV-A59, and neurotropic MHV-JHM strains were previously characterized [1,53]. The hepatotropic MHV-3 strain was provided by Dr. Julian Leibowitz (Texas A&M University). Recombinant MHV-A59 virus expresses the GFP in place of the ORF4 [54]. Cells were infected at 1 MOI by adsorption for 1 h in the basal medium. Then, excess virus inoculum was removed and cells were incubated for up to 18 h p.i. Plaque assays were performed on L2 murine fibroblasts [55]. 100% confluent monolayers of L2 cells were infected with 10-fold dilutions of samples in DMEM supplemented with 2% v/v hiFBS. After virus adsorption, cells were overlaid with a 1:1 mixture of 1.4% agarose and 2% FBS in DMEM and incubated for 48 h at 37°C with 5% CO_2_. To visualize viral plaques, infected cells were overlayed with equal parts of 1.4% agarose, 2% FBS in DMEM and 1/25 Neutral Red (Harleco, EMD Chemicals Inc., Gibbstown, NJ, USA) and incubated for 6 h at 37 °C with 5% CO_2_. 

### 3.3. Stimulation with TLR Ligands

All TLR agonists were purchased from InvivoGen (San Diego, CA, USA). The following TLR ligands were used: HKLM for TLR2 at 10^8^ cells/mL; poly I:C for TLR3 at 0.25 to 1 μg/mL; ultrapure LPS from *E. coli* for TLR4 at 5 μg/mL; and imiquimod (R837), an imidazoquinoline amine analogue of guanosine, for TLR7 at 5 μg/mL. Cells were prestimulated with the appropriate TLR ligand for 6 h, infected as necessary and coactivated during infection with the corresponding TLR agonist for up to 18 h p.i.

### 3.4. Quantification of Cytokines

Mouse IL-6 and TNF-α were quantified by ELISA (eBioscience, San Diego, CA, USA) according to the manufacturer’s instructions. Limits of detection: 7.8–500 pg/mL for IL-6 and 15.6–1000 pg/mL for TNF-α. VeriKine Mouse IFN-α and VeriKine Mouse IFN-β ELISA kits were purchased from PBL Interferon Source (ThermoScientifc, Rockford, IL, USA). Limits of detection: 15.6–1000 pg/mL for IFN-β, and 12.5–400 pg/mL for IFN-α. 

### 3.5. Titration of Anti-IFN-β Antibodies and Neutralization Assay

To titrate anti-IFN-β Ab, J774A.1 macrophages were activated with 0.25 μg/mL Poly I:C in the presence or absence of anti-IFN-β Ab at 500, 1000, or 2000 U/mL for 6 h. Rabbit polyclonal anti-IFN-β Ab were purchased from Calbiochem (EMD Chemicals Inc.). Poly I:C-triggered IFN-β with or without neutralizing Ab was assessed with a VeriKine IFN-β ELISA kit. Cell-free CM from poly I:C-activated and/or 2000 U/mL anti-IFN-β Ab-incubated macrophages was used to pretreat J774A.1 cells for 3 h. Then macrophages were infected with 1 MOI MHV-A59 by adsorption for 1h and incubated in fresh medium for up to 18 h p.i.

### 3.6. RNA isolation and Real-TimePCR

Total RNA was isolated using TRIzol^®^ reagent (Invitrogen, Carlsbad, CA, USA). Total RNA (500 ng) of was transcribed into cDNA with the Superscript II Reverse Transcriptase kit (Invitrogen), using a total reaction mix volume of 20 μL; 1.25 μL cDNA was combined with 12.5 μL of TaqMan Universal Master Mix (Applied Biosystems, Life Technologies, Carlsbad, CA, USA), 10 μL diethyl polycarbonate (DEPC)-treated water, and 1.25 μL murine TLR1 to TLR9 TaqMan Gene Expression Assays (Applied Biosystems). DNA was amplified using the Applied Biosystems 7300 Real-Time PCR machine, and cycle threshold values (C_T_) were recorded. Basal TLR1-9 mRNA levels were expressed as ΔΔC_T_ values relative to 18S rRNA [ΔΔC_T_ = ΔC_T(TLR)_ − ΔC_T(18S rRNA)_]. TLR mRNA expression levels were expressed as fold changes relative to mock values, using the variable 2^−ΔΔCT^. IFN-4α and IFN-β gene expression was quantified as above using specific pre-developed TaqMan Gene Expression Assays (Applied Biosystems). 

### 3.7. Flow Cytometry

Expression of cell surface TLR2 and TLR4, and endosomal TLR3 and TLR7 was determined using a FACSCalibur flow cytometer (BD Biosciences, San Jose, CA) and data were processed using FlowJo software (TreeStar, Ashland, OR). Cells were stained and analyzed by using a Fixation & Permeabilization kit (eBioscience) following the recommended protocols. Specific antibodies against murine TLR2, 3, 4, and 7, fluorescence-labeled secondary antibodies, isotype controls, and anti-FcrR (for blocking) were purchased from Invivogen, Imgenex (San Diego, CA), and eBioscience (San Diego, CA).

### 3.8. Fluorescence Microscopy

GFP expression was analyzed in live or 4% paraformaldehyde fixed cells. Cells were incubated with 300 nM DAPI (4',6'-diamidino-2-phenylindole dilactate; Invitrogen, Molecular Probes, Eugene, OR, USA) for nuclei stain for 3 min at RT. Images were obtained with an Olympus 1X81 motorized inverted fluorescence microscope (Center Valley, PA, USA) using Digital Microscopy software (Slidebook™ 5.0 software, Intelligent Imaging Innovations, Denver, CO). 

### 3.9. Statistical Analysis

An unpaired two-tail Student’s *t* test was used to determine statistical significance. All data were analyzed with GraphPad Prism (GraphPad Software, Inc., CA, USA) 

## 4. Conclusions

Our goal was to investigate the effects of triggering TLR2, TLR3, TLR4 and TLR7 with selected ligands on J774A.1 macrophages susceptibility to MHV infection. Our data demonstrates that triggering of TLRs with HKLM (TLR2 agonist), LPS (TLR4 agonist), and R837 (TLR7 agonist) does not affect MHV-A59, MHV-JHM, and MHV-3 production. The absence of TLR2-, TLR4-, and TLR7-mediated antiviral effects is not explained by their lack of expression or activation in J774A.1 macrophages; rather, it is based on the weak induction of type I IFN. In contrast, stimulation of macrophages with poly I:C added to the cells to trigger endosomal TLR3, induces a strong type I IFN-dependent antiviral response. Neutralization of IFN-β successfully restored the poly I:C-inhibited production of MHV-A59 in macrophages. Taken together, activation of TLR3 by poly I:C may be a successful antiviral approach against CoVs *in vivo*; its therapeutic potential as a curative drug remains to be established in future investigations. 
